# Corrigendum

**DOI:** 10.1002/ame2.12289

**Published:** 2022-10-26

In Nazar‐Zadeh et al.,^1^ the authors would like to revise the figure 1 as follows,


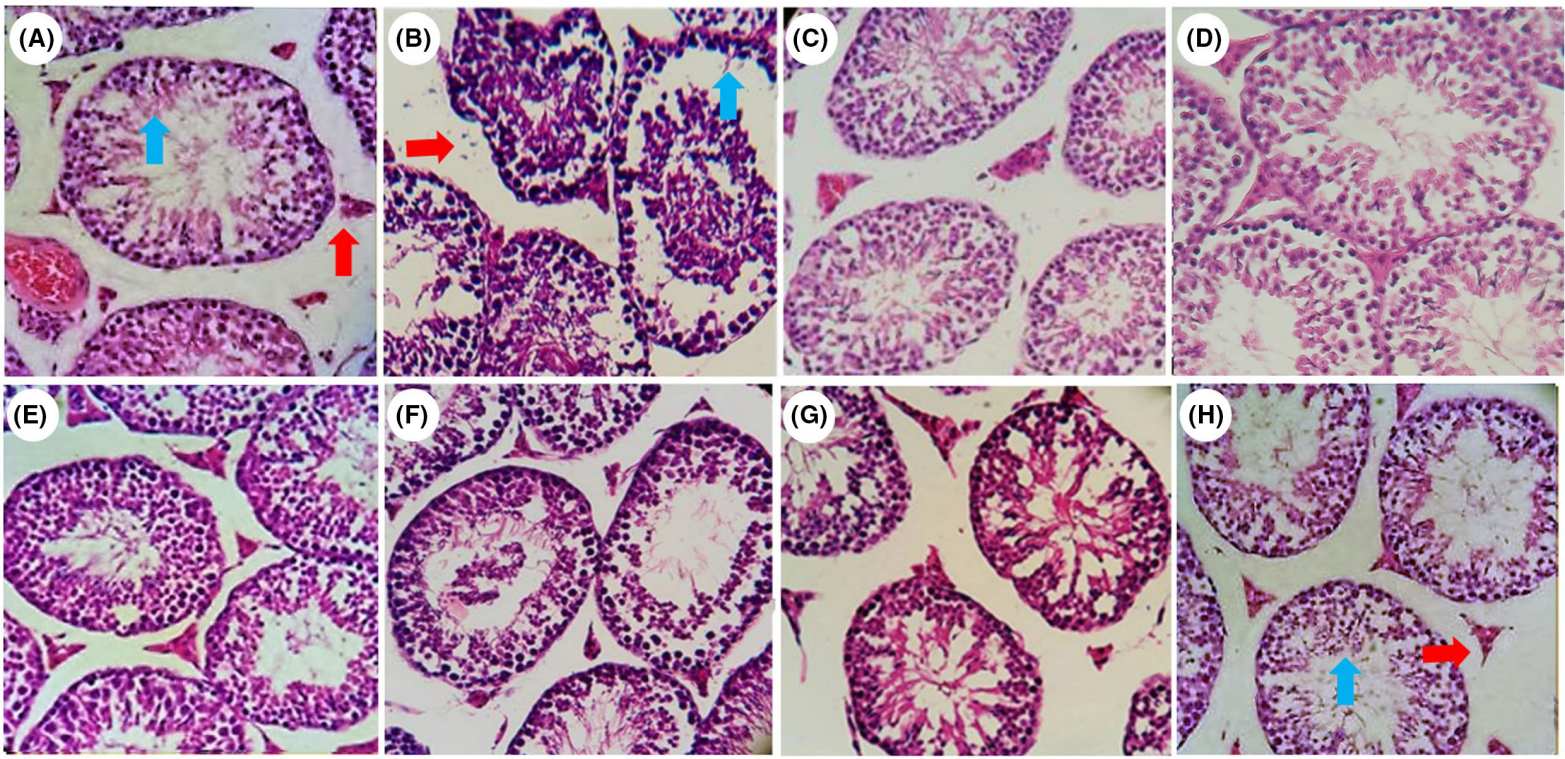


FIGURE 1 Histopathological images of testes in control and treatment groups including A: control, B: Nic, C: Roy 100 mg/kg, D: Roy 150 mg/kg, E: Roy 200 mg/kg, F: Nic + Roy 100 mg/kg, G: Nic + Roy 150 mg/kg, H: Nic + Roy 200 mg/kg. N = 6 mice in each group. Red arrow indicated the interstitial tissue in normal group (A), damaged group (B), and restored group (H). Blue arrow represented the normal germinal epithelium in control group (A), damaged dispersed germinal epithelium in Nic group (B), and repaired damaged epithelium after Roy administration (H). Nic, nicotine; Roy, royal jelly. Scale bar: 100 μm (H&E staining, 400x)

The Authors apologize for this error.
